# Model and Simulation Studies of the Method for Optimization of Dynamic Properties of Tachometric Anemometers

**DOI:** 10.3390/s18082677

**Published:** 2018-08-14

**Authors:** Paweł Ligęza

**Affiliations:** Strata Mechanics Research Institute of the Polish Academy of Sciences, 27, Reymonta str., 30-059 Cracow, Poland; ligeza@img-pan.krakow.pl

**Keywords:** mechanical anemometer, vane anemometer, cup anemometer, dynamic properties, measurement uncertainty, modelling, optimization

## Abstract

Mechanical tachometric anemometers, based on the phenomenon of the exchange of momentum between the flow and rotating measuring element, represent an important class of instruments used in flow metrology. In particular, they are used in meteorological and ventilation measurements. Mechanical anemometers with rotating measuring element are, however, known for their drawback related to their poor dynamic properties resulting from relatively large dimensions and mechanical inertia of the measuring element. In these instruments, the phenomenon of overestimating the measured average velocity caused by the inertia of the rotor takes place. Optimization of the dynamics of the measurement process, as well as the estimation and minimization of the measurement uncertainty, can be performed based on mathematical model of anemometer. In this study, a new, original concept of optimization of dynamic properties of tachometric anemometers is proposed, and the results of model and simulation studies are presented. The new concept of measuring instrument is based on the use of feedback and active control of the rotor. The new method was tested using model research, where two types of flow velocity excitations were applied: sinusoidal and rectangular. The tests carried out showed that the developed method allows for minimization of the dynamic uncertainty of the measurement and minimizes the phenomenon of average flow velocity overestimation occurring in time-varying flows. It has been shown that the use of optimization system allows for approximately tenfold reduction of the error of average velocity measurement in the case of pulsating flows. In addition, the optimization systems allow for anemometer’s transmission bandwidth to be extended about a hundred times. This creates new application possibilities for these instruments and allows for a large reduction of measurement uncertainty.

## 1. Introduction

Mechanical anemometers with rotating measuring element (i.e., impeller, rotor, or turbine wheel) represent an important class of instruments used for measuring the flow velocity of fluids. They are based on the phenomenon of the exchange of momentum between the flow and the rotating measuring element. These instruments are widely applied in flow metrology, and their main area of application covers the metrological and ventilation measurements. An interesting review of development of this kind of anemometer can be found in [1]. Based on their construction, anemometers with rotating measuring element may be divided into two basic groups. The first one involves vane (or propeller) anemometers, in which the measuring element rotates itself in a plane perpendicular to measured velocity vector. The second one, on the other hand, includes the cup anemometers, in which the measuring element rotates in a plane parallel to measured flow velocity vector.

Mechanical tachometric anemometers offer a series of advantageous metrological features. They have approximately linear characteristics, allow measurements of a wide range of velocities, and are less sensitive to changing physical parameters of the flowing medium. On the other hand, due to their relatively large dimensions and mechanical inertia of rotating measuring element, these anemometers tend to average the measurement results: one in time, and the other in space. Even though it may be a beneficial property in many situations, measurements in heterogeneous velocity fields, as well as in time-varying flows, may be burdened with significant errors. An unfavorable phenomenon of overestimation of the result of average velocity measurement can thus often be seen. Analysis of this phenomenon, and the development of methods facilitating its reduction, can be performed based on mathematical model of such an anemometer. There is a huge amount of literature dedicated to development of theoretical models of anemometers with rotating measuring element, as well as to their verification.

The study by Patterson [2] is one of the first scientific reports dealing with the issue of the construction and dynamics of three cup anemometer. Another fundamental work by Ower [3] provides an equation of motion for vane anemometer, and points to the overestimation of the measured average value due to the rotor’s inertia. In subsequent studies, the authors considered an equation of motion of the rotor. The moments of forces related to the exchange of momentum, as well as to the friction, were taken into account. The study [4] reports the analysis of unsteady response of a turbine anemometer, while the study [5] is dedicated to measurements of pulsating flows. An interesting overview of achievements in this area is presented in the study [6]. Study [7] concerns the measurement of turbulent flows. Issues of dynamics of anemometer designed for measurements of fluids are considered in study [8]. Analysis of the response of anemometer to acoustic fluctuations is discussed in the study [9]. The effect of air density on anemometer calibration is the topic of work [10]. In [11], an attempt to apply the artificial neural networks for modelling the cup anemometer was undertaken. In [12], the effect of environment viscosity on anemometer’s operation is analyzed by means of the CFD analysis. Study [13], on the other hand, is dedicated to dynamic measurements in pulsating flows. A starting point in these articles is provided by the second law of dynamics for the rotation of anemometer’s rotor, taking into account the total moment of forces acting on this rotor. Such a model describes the dynamic dependence of the velocity indicated by the anemometer on the actual velocity. Model parameters may be calculated based on directly measurable physical quantities, or may be experimentally determined.

The author of study [14] has however shown that a simple model of anemometer based on the motion equation of the rotor [3,4,5,6,13] does not allow for sufficiently accurate modelling of all dynamic phenomena. Furthermore, extension of such a model [10,11,12] results in increased complexity of its structure, and to an increased number of model parameters, the determination of which requires complex experimental research. Although these models provide better approximations of the actual object, they are, however, less useful in the process of analysis of dynamic states of anemometer, as well as in estimation and minimization of the measurement uncertainty.

For this reason, an alternative model of anemometer, based on the balance of power, is proposed in paper [14] as suitable for the analysis of dynamic phenomena. Based on this model, it is possible to conduct relatively accurate measurements, optimize the dynamics of a measurement process, as well as to estimate and minimize the measurement errors. One example of such a measurement may be the traversing of time-varying velocity fields in order to determine the volumetric flow [15,16]. Due to the large inertia of anemometers with rotating measuring element, measurements with this kind of instrument must be supported by other instruments, including hot-wire anemometers [17,18].

In the latest literature, there is a lack of new solutions significantly improving the dynamic properties of tachometric anemometers. The few new concepts may include the proposal to use axial force to reduce dynamic errors in the vane anemometer [19,20]. The newest work carried out in the field of anemometer construction and development focuses mainly on the study and optimization of the shape of rotor elements [21]. In this paper, the influence of anemometer rotor shape parameters, such as the cups’ front area or their center rotation radius, on the anemometer’s performance, was analyzed. The development of instruments is also associated with new methods of analysis and processing of the output signal from the tachometric anemometer [22]. The Fourier analysis of the signal is used here. Another issue related to the development of mechanical anemometer constructions are studies on the durability of instruments in various climatic conditions and the detection of failures [23]. However, there are no new solutions allowing for radical improvement of dynamic parameters of tachometric anemometers. Therefore, the harmful phenomenon of overestimating the mean value of measurements in fast-changing flows is one of the main sources of dynamic errors.

Our article analyzes a completely new, original method of solving this problem by applying active rotor control. The work is focusing on reduction of the phenomenon of overestimating the measured average velocity. In this study, we propose a principle of the new method for optimization of dynamic properties of an anemometer with rotating measuring element. The solution under discussion is a subject of patent [24]. The proposed original method, together with respective system solution, are presented, as well as results of model analysis aimed to compare the anemometer equipped with optimization system to the one without it. The concept of the method of dynamic properties optimization is illustrated using the vane anemometer. Nevertheless, it seems reasonable to state that the hereby presented considerations and method may successfully be adapted also in the case of cup anemometers.

## 2. Mathematical Model of Vane Anemometer Based on the Balance of Power

Author of the study [14] proposes an original mathematical model of vane anemometer based on the balance of power. Such a balance is based on the fact that the change of kinetic energy of an anemometer’s rotor equals the difference in kinetic energy of the stream of medium entering and exiting the active cross-sectional area of the rotor. Developing the model based on the power balance, author assumed the conservative nature of the process, not including friction forces. Thanks to this, he received a simple and elegant, simplified model of the phenomenon. This model can be used for initial analysis of dynamic phenomena. Taking into account the friction forces will result in a significant expansion of the model [7,23]. The author assumes a conservative character of the process and the homogeneous velocity field measured in a medium of the density *ρ*. It is assumed that the rotor is equipped with wings inclined to the flow direction at an angle *α*, the distance between aerodynamic center of the wings and the axis of rotation is *R*, cross-sectional area of interaction between the flow and the rotor is *S*, the moment of inertia of the impeller with respect to the axis of rotation is *J*, and the angular velocity of rotor is *ω*.

The balance of power associated with the exchange of kinetic energy between the flowing medium and the anemometer’s rotor may thus be written as

(1) $$\frac{dE_{R}}{dt}=\frac{dE_{I}}{dt}-\frac{dE_{O}}{dt},$$

where

*E_R_*—Kinetic energy of the rotation of rotor,
*E_I_*—Kinetic energy of the mass of medium entering the rotor,
*E_O_*—Kinetic energy of the mass of medium leaving the rotor.

When building the model [14], the author has adopted an original assumption according to which the flowing medium leaves the cross-sectional area of the rotor *S* with the average velocity of *V*, which is equal to

(2) $$V=\frac{\omega R}{\mathrm{tg}\alpha }.$$

This postulate reflects, in fact, the action of an anemometer’s rotor, seen as a kind of turbine. If the measured velocity *v* of medium flowing towards the rotor is less than *V*, the medium is being sucked up by the turbine, while if the velocity *v* is greater than *V*, the flow is being suppressed. If both these velocities are equal to each other, we have a steady state in which no energy exchange between the rotor and flow takes place. According to an adopted assumption, the medium mass entering and exiting the active cross-sectional area of the anemometer during the time interval d*t* equals

d*m* = *ρsv* d*t*. (3)

In light of (3), the balance of power (1) may then be written as

(4) $$J\omega \frac{d\omega }{dt}=\frac{\rho SV_{V}{}^{2}}{2}-\frac{\rho SVV^{2}}{2}\;$$

Using the dependence (2) as an anemometer’s output equation describing the relationship between the angular velocity of the rotor *ω* and the value of velocity *V* indicated by the anemometer, Equation (4) will give us the mathematical model of the vane anemometer, given the adopted assumptions, in the following form:

(5) $$c\frac{dV}{dt}=-V^{2}+v^{2},$$

where $c=\frac{2Jtg^{2\alpha }}{\rho R^{2}S}$ is the only parameter of the model with the dimension of length. According to Equation (5), the velocity *V* indicated by the anemometer in the steady state is equal to the actual measured velocity *v*. Following an appropriate correction of Equation (2), the hereby presented model of the vane anemometer (5) may be adopted to describe the cup anemometer. It should be noted that Equation (5) is a non-linear equation, in contrast to linear models under consideration in studies [3,4,5,6,13], and provides better reflection of actual physical process of anemometer’s operation [14]. In the next part of the study, the concept of the method and an example solution of the system for optimization of dynamic properties of an anemometer with rotating measuring element are discussed. Based on model (5), comparative modelling—comparing the anemometer equipped with optimization system to the one without it—is also presented.

## 3. Principles of the Method for Behavior Enhancement of Anemometer’s Dynamic Properties

Mechanical anemometers with rotating measuring element are characterized by a drawback consisting in their poor dynamic properties resulting from relatively large dimensions and mechanical inertia of the measuring element. Time required to stabilize the steady state of the rotor following the change of flow velocity is significant, with this time being shorter in the case of rotor acceleration, and longer in the case of its deceleration. As a result of this phenomenon, results of measurement of time-varying flows require complex interpretation, and are burdened with considerable uncertainty. In these anemometers, the average flow velocity measured in time-varying flows may be overestimated by even as many as several percent. Study [19] presents the research on the possibility of usage of the axial force of vane anemometer to compensate for this phenomenon. The author of that study presents the preliminary results of experimental and model analysis, as well as of practical applicability of this method.

**Figure 1:**
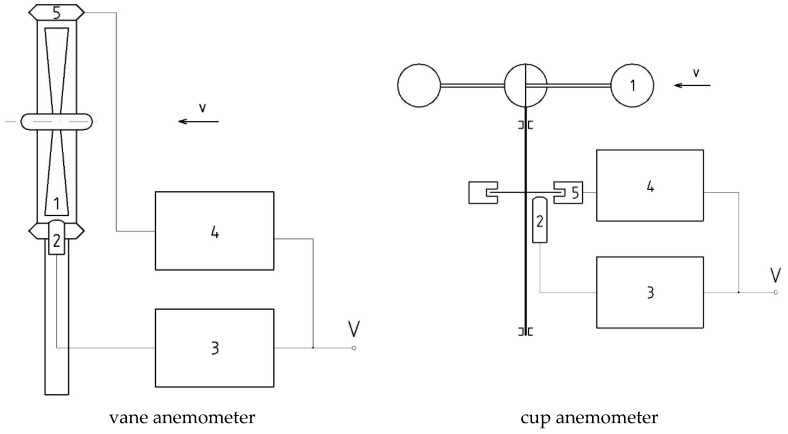
A system for optimization of dynamic properties of anemometers with rotating measuring element.

Alternatively, an original solution is presented in this paper. Figure 1 presents the scheme of the proposed system for optimization of dynamic properties of an anemometer with rotating measuring element.

The principle of operation of the system will be explained on the example of the vane anemometer. The improved system consists of anemometer’s rotor 1 placed in the measured flow with the velocity *v*, the rotor speed sensor, 2, a system converting the rotational speed of rotor to output signal, 3, electronic system supporting the rotor’s acceleration or deceleration, 4, and an assembly of electromagnets, 5. By exchanging the momentum between the measured flow *v* and the rotating measuring element, the rotor gains rotational speed, which is then the measure of the flow velocity. The rotor is made of a conductive material or a material interacting with the magnetic field. Its rotational speed is measured by sensor, 2, and subsequently converted in block, 3, to output signal *V*, being the measure of flow velocity. At the same time, the system supporting the rotor’s acceleration or deceleration, 4, processes this signal and converts it to a signal controlling the assembly of electromagnets, 5, placed at the circumference of the rotor. The supporting system, 4, operates sequentially according to a given algorithm, controlling the electromagnet assembly such that the magnetic field and eddy currents generated in the rotor or by magnetic interaction produce torque, enabling increase or decrease of rotor’s angular velocity.

The process of optimization of dynamic properties of an anemometer with rotating measurement element is implemented in block 4, sequentially, in two stages. In the first stage, the d*V/*d*t* derivative of the measurement signal *V* is calculated to determine whether the flow-rotor interaction causes an increase or decrease in rotational speed of the rotor. In the second stage, a signal in the function of the intensity of measured signal *V* and derivative d*V*/d*t* is generated to the electromagnet assembly, 5. If the derivative is positive, the electromagnet assembly, 5, generates an instantaneous moment of force which accelerates the rotor, 1, and if the derivative is negative, it creates a moment of force to slow down the rotor, 1. Then, the signal generating the moment of force is being turned off, and the process returns to the first stage and starts, cyclically, all over again. In this way, the process of keeping the rotational speed of the rotor up with changes of flow velocity is accelerated, and the dynamic properties of the anemometer become optimized.

A simplified operational mode offering only the function of rotor decelerating is also available. It allows for the rotor decelerating time to be shortened, and thus, for the rotor acceleration and deceleration times to be equalized. The method and system for optimization of dynamic properties of an anemometer with rotating measuring element allow reduction of the time required for an anemometer to respond to changes in velocity signal, as a result of which, similar reaction times can be obtained during both the rotor’s acceleration and deceleration. This minimizes the dynamic uncertainty of the measurement and minimizes the phenomenon of average flow velocity overestimation occurring in time-varying flows.

The proposed concept and system for optimization are further discussed using the vane anemometer as an example. Similar solutions, Figure 1, can be used in the case of cup anemometer by placing a suitable disk interacting with the electromagnet assembly on the rotor axis. The electromagnetic accelerating–decelerating unit can also be used for measurement of rotational speed of the rotor, as well.

## 4. A Model Study of the Method for Optimization of Anemometer’s Dynamic Properties

Analysis of the hereby proposed method and system for optimization of dynamic properties of an anemometer with rotating measuring element was performed on the basis of computer simulation. To this end, the MATLAB interactive science and engineering environment was used. Steady state of the response of the anemometer with or without optimization system to sinusoidal or rectangular excitation velocity signal was analyzed.

Based on the model (5), comparative model analysis aimed to compare the vane anemometer equipped with optimization system to the one without it was performed. Physical parameters of an actual anemometer provided in [20] were assumed. These parameters are summarized in Table 1.

The model (5) was introduced with a function *u* allowing the simulation of the process of decelerating of accelerating the rotor by means of the magnetic field:

(6) $$c\frac{dV}{dt}=-V^{2}+v^{2}+u,$$

where

*u* represents the output of the feedback system, and it means the additional impact on the rotor provided by the optimization system, with the dimension of squared velocity.

In the simulation, this function was assumed to be in the following form:

(7) $$u=\frac{h_{i}^{2}}{\Delta t}\frac{V_{I}\prime }{V_{I}},$$

where

*V_I_*—the velocity determined in the first stage of the optimization cycle,
$V_{I}^{\prime }$—derivative of the velocity determined in the first stage of the optimization cycle,
Δ*t*—duration of the second stage of the optimization (excitation generation),
*h_i_*—the parameter of acceleration or deceleration of the rotor with the dimension of length.

The form of the function was selected heuristically on the basis of previously performed analyses and preliminary results of model analysis. The *h_i_* parameter in the first stage of the optimization process takes the value of zero, whereas in the second stage, it has a selected constant value. In performed model studies, it was assumed that *h_i_* = 1 m. It is also possible to have the value of the parameter *h_i_* being dependent on the sign of the derivative, or two different values for decelerating and accelerating.

Two types of velocity excitations were used in the simulation testing—the sinusoidal excitation in the form

*v*_0_ = *v_m_*(1 + *k sin*(2π*ft*)), (8)

and the rectangular excitation

*v*_0_ = *v_m_*(1 + *k sgn*(sin(2π*ft*))), (9)

where

*v_m_*—the average value of excitation,
*k*—a coefficient in the range [0, 1] modulating the average value,
*f*—modulation frequency of the excitation.

**Figure 2:**
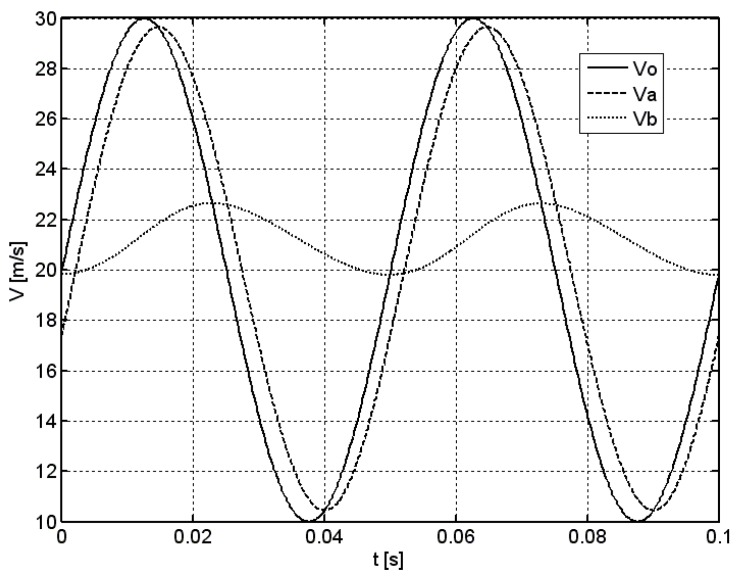
The time course of the response of vane anemometer with or without the optimization system to a sinusoidal excitation, *v_m_* = 20 m/s, *k* = 0.5, *f* = 20 Hz.

Figure 2 presents an example of two periods of the simulation in the case of sinusoidal excitation for average velocity of *v_m_* = 20 m/s, modulation factor of *k* = 0.5, and the frequency of *f* = 20 Hz. Solid line denotes the time course of the excitation *v*_0_, while the dashed and dotted lines denote the response of an anemometer with and without the optimization system *v_a_* and *v_b_*, respectively.

In the case of standard vane anemometer without the optimization system, a significant phase shift and decrease of amplitude of its response *v_b_* can be seen when compared to excitation *v*_0_. In addition, the average value of the response is increased. Contrary to this, the response of an anemometer equipped with the optimization system *v_a_* is similar to the excitation signal, which confirms the beneficial effects of such an optimization system. Its transmission bandwidth is thus considerably extended compared to the one of standard anemometer. In model studies, more than a hundredfold extension of the anemometer’s bandwidth was observed, following the employment of the optimization system. Such a result seems to be, however, difficult to obtain in actual measurement system, due to its physical restrictions. Nevertheless, it needs to be experimentally proven.

**Figure 3:**
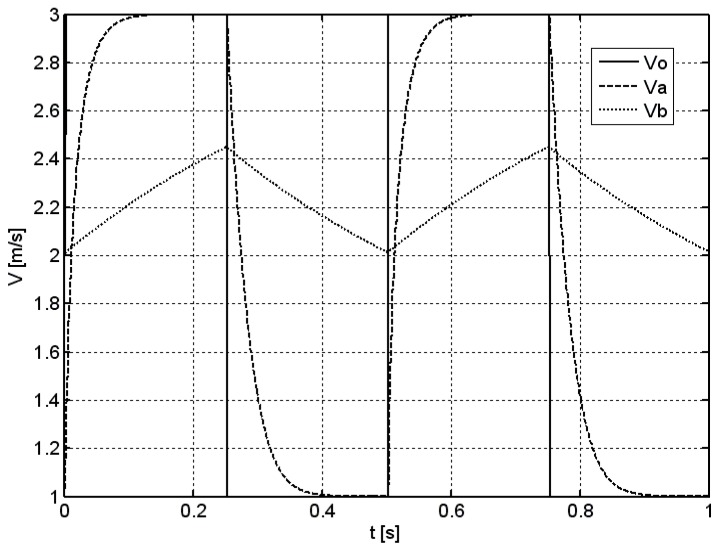
The time course of the response of vane anemometer with or without the optimization system to a rectangular excitation, *v_m_* = 2 m/s, *k* = 0.5, *f* = 2 Hz.

Figure 3 presents an example of two periods of the simulation in the case of rectangular excitation for an average velocity of *v_m_* = 2 m/s, modulation factor of *k* = 0.5, and frequency of *f* = 2 Hz. As in the previous case, a solid line denotes the time course of the excitation *v*_0_, while the dashed and dotted lines denote the response of anemometer with and without the optimization system *v_a_* and *v_b_*, respectively.

Observed effects are similar to those obtained in the case of sinusoidal excitation.

**Figure 4:**
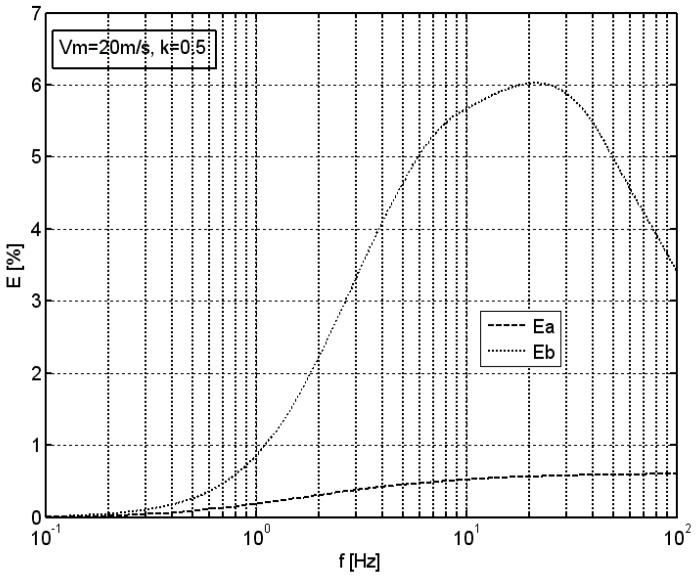
Relative error of the average velocity measured by an anemometer with (*Ea*) or without (*Eb*) the optimization system in the function of the frequency of sinusoidal excitation, *v_m_* = 20 m/s, *k* = 0.5.

In order to perform a parametric evaluation of the hereby presented optimization method and the system, simulations were conducted in which the relative error *E* of the average velocity measured by the anemometer, either with or without the optimization system, was calculated. Figure 4 presents the values of this parameter against the frequency of sinusoidal excitation, given the average velocity of *v*_m_ = 20 m/s and the modulation factor of *k* = 0.5. In this, as well as in the following figures, the dashed line shows the error *Ea* of the anemometer with the optimization system, while the dotted line shows the error *Eb* of the anemometer without such optimization system. The frequency is presented in logarithmic scale.

**Figure 5:**
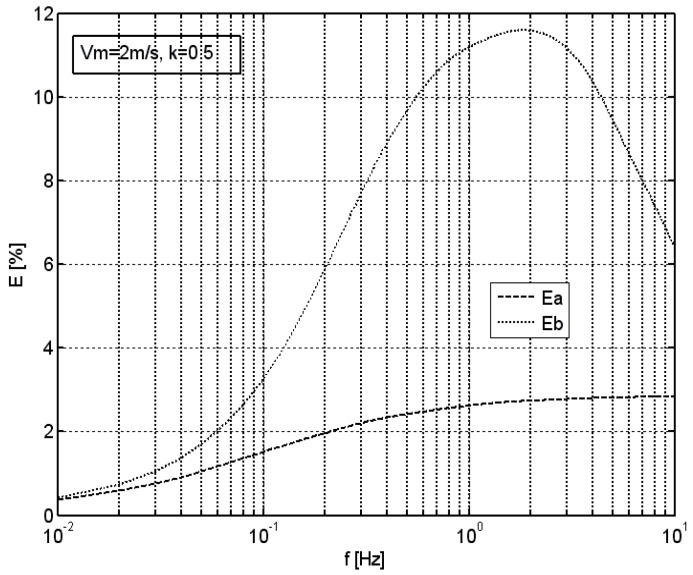
Relative error of the average velocity measured by an anemometer with (*Ea*) or without (*Eb*) the optimization system in the function of the frequency of rectangular excitation, *v_m_* = 2 m/s, *k* = 0.5.

Figure 5 shows an analogous graph for the rectangular excitation given the average velocity of *v_m_* = 2 m/s, and the modulation factor of *k* = 0.5.

In both cases, standard vane anemometer without the optimization system presents an increased relative error of the average velocity measurement, which increases with increasing frequency all the way up to a certain characteristic frequency. Performed analyses show that the value of this frequency is approximately the linear function of the average velocity of the excitation. For parameters used in the simulation, this frequency was 2 Hz in the case of *v_m_* = 2 m/s, and 20 Hz in the case of *v_m_* = 20 m/s. Above this frequency, the relative error of the average velocity measurement decreases, which is related to strongly decreasing amplitude of anemometer’s response. In the case of an anemometer equipped with the optimization system, an increase in the relative error of the measurement of average velocity is also observed, but its magnitude is considerably lower. In the case of sinusoidal excitation, the optimization system of the hereby adopted structure enables an approximately tenfold reduction of the relative error of the average velocity measurement in the range up to the characteristic frequency. In the case of rectangular excitation, such a reduction is approximately fourfold. This is related to the rectangular signal spectrum, which contains components of higher frequencies.

**Figure 6:**
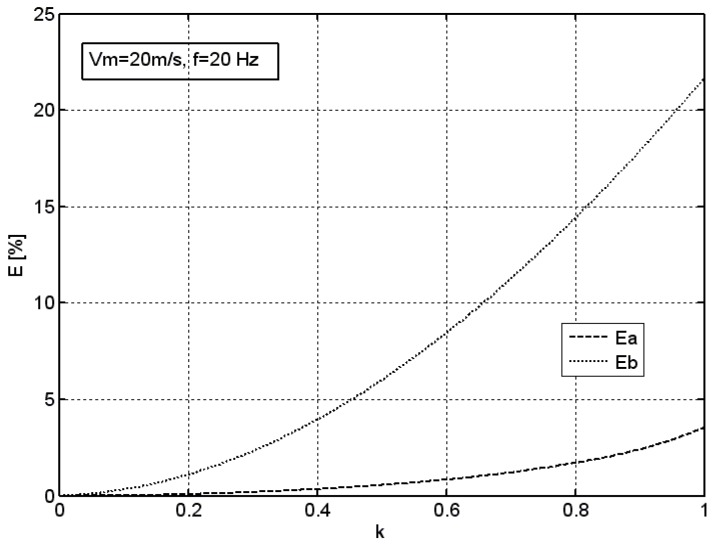
Effect of the coefficient of modulation *k* of sinusoidal excitation on the error of average velocity measurement *E* for both measuring systems, *v_m_* = 20 m/s, *f* = 20 Hz.

In order to determine the influence of the amplitude of an excitation component on the error of average velocity measurement for both measuring systems, simulations using the sinusoidal excitation and variable coefficient of modulation of excitation *k* was conducted. Results of this simulation for an average velocity of *v_m_* = 20 m/s and frequency of *f* = 20 Hz are presented in Figure 6.

Performed simulations indicate that the optimization system allows for tenfold reduction of the error of average velocity measurement basically in the whole range of the modulation coefficient *k* = 0...1.

**Figure 7:**
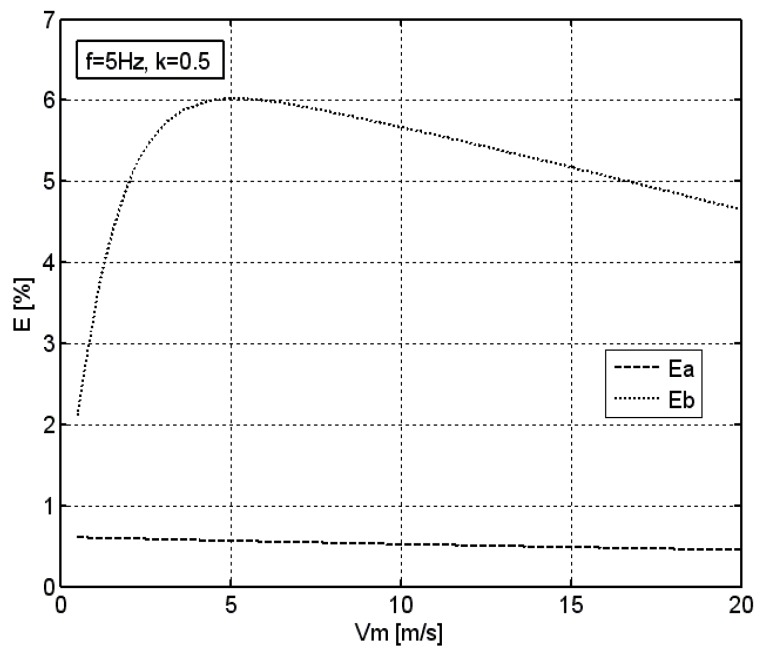
The effect of average velocity of sinusoidal excitation *v_m_* on the relative error of its measurement *E* for both measuring systems, *k* = 0.5, *f* = 5 Hz.

In order to determine the influence of the average velocity of the excitation on the error of its measurement by means of both the measuring systems, simulations using the sinusoidal excitation were conducted. Results of such simulations, given the average velocity *v_m_* being in the range from 1 to 20 m/s, the coefficient of modulation *k* = 0.5, and the frequency of *f* = 5 Hz, are presented in Figure 7.

In the case of an anemometer without the optimization system, the relative error of the average velocity measurement increases with increasing velocity up to a certain value, and then decreases. In the examined case, given the frequency of excitation of *f* = 5 Hz, a maximum of this error with the value of 6% occurs at the velocity of *v_m_* = 5 m/s, which is in line with the above-provided conclusion on the linear dependence between the excitation frequency and the average velocity at which the maximum error occurs.

In the case of an anemometer equipped with the optimization system, the effect of the average velocity of sinusoidal excitation *v_m_* on the relative error of its measurement is negligible. Given the coefficient of modulation of *k* = 0.5 and the frequency of *f* = 5 Hz, this error is maintained at the level of around 0.5% in the whole range of the average velocity.

## 5. Conclusions

In this study, the new method for optimization of dynamic properties of an anemometer with rotating measuring element using active rotor control was proposed. The study discussed the implemented mathematical model of an anemometer based on the balance of power and presented the main principles of the optimization method, as well as the methodology of model testing. Theoretical analysis and simulations of a new method and system for optimization of dynamic properties of an anemometer with rotating measuring element were performed. The developed method allows for minimization of the dynamic uncertainty of the measurement and minimizes the phenomenon of average flow velocity overestimation occurring in time-varying flows.

Model testing confirmed that in tachometric anemometers, the average flow velocity measured in time-varying flows may be overestimated by even as many as several percent. It has been shown that the use of an optimization system allows for an approximately tenfold reduction of the error of average velocity measurement in the case of pulsating flows. In addition, the optimization systems allow for anemometer’s transmission bandwidth to be about a hundred times extended. Standard tachometric anemometers enable tracking of flow velocity changes only for very slow fluctuations. It can be estimated that for devices with low rotor inertia, a frequency bandwidth of around 0.1 Hz can be obtained [7,8,20]. The tests carried out indicate that the application of the active control system allows one to obtain a frequency bandwidth of approximately 10 Hz. This creates new application possibilities for these instruments, and allows for a large reduction of measurement uncertainty. Simulation studies have also allowed us to determine the effect of individual experimental parameters on the efficiency of an optimization system.

The results indicate that the method may prove useful in optimization of dynamic characteristics of mechanical tachometric anemometers. This, however, involves additional construction components which need to be introduced to anemometers’ construction, as well as additional extension and development of the software controlling the anemometer’s operation. It renders its construction more complex, increases the power consumption, as well as the price of the measuring device. The rotor speed measurement sensor must provide a very fast angle measurement with high resolution. Such measurement can be obtained by using an integrated circuit, on axis magnetic rotary position sensor from the AMS company (Unterpremstätten, Austria) [25]. Such a sensor has a 14-bit angle resolution per one revolution, and 250 ns sampling rate. The rotor acceleration and deceleration device must have sufficient maximum torque and control range. For example, a disc magnet micro stepper motor from the Portescap company (West Chester, PA, USA) [26], with 60 steps per revolution, may be used. The axis and bearing system of such a motor can be used as the rotor axis of the anemometer. The control microprocessor system, together with the software, is a less critical element, because the currently available microcontrollers of many companies meet the parameters required in the proposed solution, with an excess.

Nevertheless, the hereby proposed solution may be successfully implemented in a certain class of instruments, especially those in which the minimizing of dynamic measurement errors is of utmost importance [15,16]. Therefore, further research is necessary, especially in the field of expanded model and experimental tests and prototype development.

## Figures and Tables

**Table 1:** Physical parameters of vane anemometer.

Parameter	Designation	Value	Dimension
density of flowing medium (air)	*ρ*	1.293	kg/m^3^
mean rotor radius	*R*	34.75 × 10^−3^	m
active cross-sectional area of the rotor	*S*	4687 × 10^−6^	m^2^
inclination angle of vanes	*α*	45	°
moment of inertia of the rotor	*J*	8.18 × 10^−6^	kg m^2^
